# Sphingosine regulates the NLRP3-inflammasome and IL-1**β** release from macrophages

**DOI:** 10.1002/eji.201142079

**Published:** 2011-12-27

**Authors:** Nadia M Luheshi, James A Giles, Gloria Lopez-Castejon, David Brough

**Affiliations:** Faculty of Life Sciences, University of ManchesterManchester, UK

**Keywords:** Caspase-1, Inflammasome, Inflammation, Interleukin-1, Sphingosine

## Abstract

Interleukin-1β (IL-1β) is a pro-inflammatory cytokine that regulates inflammatory responses to injury and infection. IL-1β secretion requires the protease caspase-1, which is activated following recruitment to inflammasomes. Endogenous danger-associated molecular patterns (DAMPs) released from necrotic cells activate caspase-1 through an NLRP3-inflammasome. Here, we show that the endogenous lipid metabolite sphingosine (Sph) acts as a DAMP by inducing the NLRP3-inflammasome-dependent secretion of IL-1β from macrophages. This process was dependent upon serine/threonine protein phosphatases since the PP1/PP2A inhibitors okadaic acid and calyculin A inhibited Sph-induced IL-1β release. IL-1β release induced by other well-characterized NLRP3-inflammasome activators, such as ATP and uric acid crystals, in addition to NLRC4 and AIM2 inflammasome activators was also blocked by these inhibitors. Thus, we propose Sph as a new DAMP, and that a serine/threonine phosphatase (PP1/PP2A)-dependent signal is central to the endogenous host mechanism through which diverse stimuli regulate inflammasome activation.

## Introduction

Inflammation is a crucial host response required for the clearance of pathogens and for the repair of injured tissue. However, inflammation that occurs in the absence of an infection, but in response to an injury, so-called sterile inflammation, may promote further tissue damage due to the production of toxic mediators and proteases by recruited immune cells [1]. As such, sterile inflammation contributes to the worsening of many diseases, including ischaemic stroke, gout, atherosclerosis and diabetes [1,2]. The pro-inflammatory cytokine inter-leukin-1b (IL-1b) has been identified as a key mediator of sterile inflammatory responses and is thus an attractive therapeutic target in the diseases to which sterile inflammation contributes [3].

In vitro, IL-1b is produced in the macrophage cell cytosol as a precursor in response to pathogen-associated molecular patterns (PAMPs) such as LPS acting via Toll-like receptor 4 (TLR4) [4]. This is generally referred to as a 'priming step' and is required prior to the subsequent cytokine release. The priming stimulus in vivo during sterile inflammatory responses is not clear although it has been suggested that several danger-associated molecular patterns (DAMPs), endogenous molecules released by dead cells or that are modified during disease, can act via the same receptors as PAMPs [5]. The secretion of IL-1β from a primed macrophage depends upon the formation of inflammasomes; large molecular scaffolds containing cytosolic pattern recognition receptors, adaptor proteins and caspase-1. Of these, the best characterized and most relevant to sterile inflammatory responses is formed by the pattern recognition receptor NOD-like receptor pyrin domain containing 3 (NLRP3) [6,7]. When NLRP3 senses DAMPs it recruits apoptosis-associated speck-like protein containing a caspase recruitment domain (ASC), which in turn recruits caspase-1 causing its activation [6]. Caspase-1 then processes pro-IL-1β to a mature form that is rapidly secreted from the cell [8].

NLRP3 is activated by structurally diverse PAMPs and DAMPs, and it is thought that these converge on an endogenous signal to cause activation [9,10]. One of these endogenous signals is suggested to be the rupture of lysosomal membranes and the activity of lysosomal proteases such as cathepsin B [10]. Multiple DAMPs are reported to activate NLRP3 inflammasomes via lysosomal destabilization and cathepsin activation [11–13]. We hypothesized that endogenous molecules that influence lysosomal stability may, under conditions of cellular stress, act as DAMPs.

Sphingosine (Sph) is an endogenous lipid mediator and its levels are modulated during cell signalling [14], and elevated during disease [15–17]. Sph accumulates in, and destabilizes lysosomes causing cell death [18,19]. We report here that Sph induced an NLRP3-dependent activation of caspase-1 and IL-1β secretion from LPS-primed macrophages in vitro. In an in vivo model of peritonitis, the water-soluble Sph analogue FTY720 induced IL-1β release and neutrophil influx into the peritoneal cavity. The DAMP effects of Sph were independent of lysosomal destabilization but rather, dependent upon serine/threonine phosphatases PP1/PP2A. Subsequent experiments revealed that a PP1/PP2A-dependent mechanism represents an endogenous signal upon which structurally diverse DAMPs converge in the activation of multiple inflammasomes.

## Results

### Sph induces IL-1β release from primed macrophages

**Fig 1:**
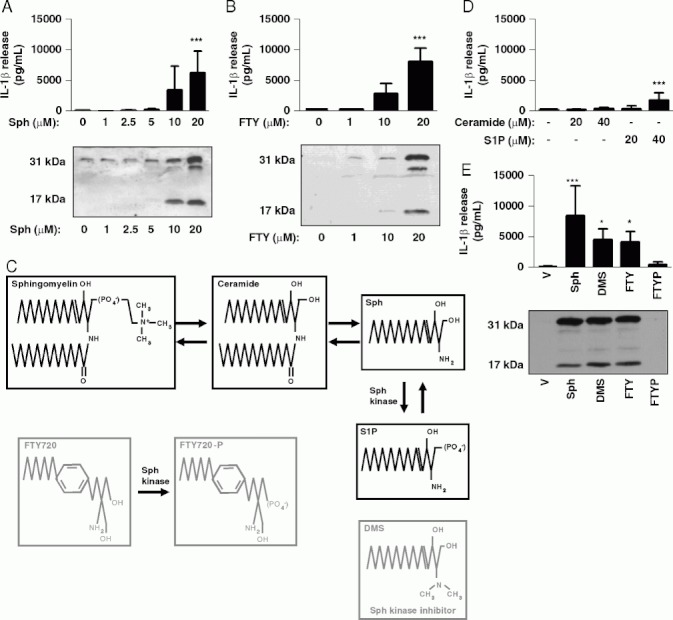
Sphingosine induces IL-1 release from LPS-primed macrophages. (A, B) LPS-treated (1 mg/mL, 2h) murine peritoneal macrophages were incubated for 1 h with the indicated concentration of (A) sphingosine (Sph) or (B) the Sph analogue FTY720. IL-1β in the supernatants was quantified by ELISA (top). Processing of pro-IL-1β (band at 31 to 17 kDa) was determined by western blot (bottom). (C) The structures of key mediators in the sphingolipid pathway are shown, and the structures of the Sph analogue FTY720, the S1P analogue FTY720-P and the sphingosine kinase inhibitor DMS are provided for comparison. (D, E) LPS-treated peritoneal macrophages were also incubated with the indicated concentrations of (D) ceramide or S1P or (E) 20 μM Sph, DMS, FTY720 and FTY720-P (FTYP) for 1h. IL-1β was quantified in supernatants by ELISA and pro-IL-1β processing to mature IL-1β was determined by western blot (E bottom). Data are shown as the mean 7 1 SD of three experiments. ***p<0.001, *fx0.05. Differences between groups were identified using one-way ANOVA with post-hoc Bonferroni multiple comparison test. Blots are representative of three experiments.

Initially, we investigated whether Sph could induce the secretion of IL-1 from LPS-primed murine peritoneal macrophages. Incubation of LPS-primed macrophages with Sph (20 μM, 1 h) induced significant release of mature IL-1β (Fig. 1A). FTY720 (FTY, fingolimod), an Sph analogue derived from a fungal metabolite, and that is approved for the treatment of multiple sclerosis [20], also induced a significant release of mature IL-1β when used at an equivalent concentration (Fig. 1B). In vivo, FTY720 is phosphoryl-ated to become an analogue of sphingosine-1-phosphate (S1P), and acts as a functional antagonist at S1P receptors, exerting an immunosuppressive effect by causing lymphocyte sequestration in lymph nodes [20]. However, at higher doses, non-phosphorylated FTY720, like Sph, is pro-apoptotic and inhibits tumour growth in animal models [21,22].

Sph is one of the bioactive sphingolipid group that, collectively, have pleiotropic effects on cellular signalling and function [23]. Since exogenous Sph addition will alter the levels of upstream and downstream sphingolipid mediators (Fig. 1C), we sought to determine whether these could be partly responsible for the effects of Sph on IL-1 release. Ceramide, the metabolite immediately preceding Sph production (Fig. 1C), had no effect on IL-1β secretion, even at high concentrations (Fig. 1D). S1P, which is immediately downstream of Sph (Fig. 1C), induced some IL-1β secretion, but at much higher concentrations (40mM, Fig. 1D). Like S1P, at 20 μM FTY720-P (FTYP) failed to stimulate IL-1β secretion (Fig. 1E). Dimethyl-sphingosine (DMS) an Sph analogue that blocks conversion of Sph to S1P by inhibiting Sph kinase (Fig. 1C) [24], also induced significant mature IL-1β secretion (Fig. 1E), strongly suggesting that Sph is the active metabolite.

### Sph-induced IL-1β release is dependent upon the NLRP3 inflammasome

**Fig 2:**
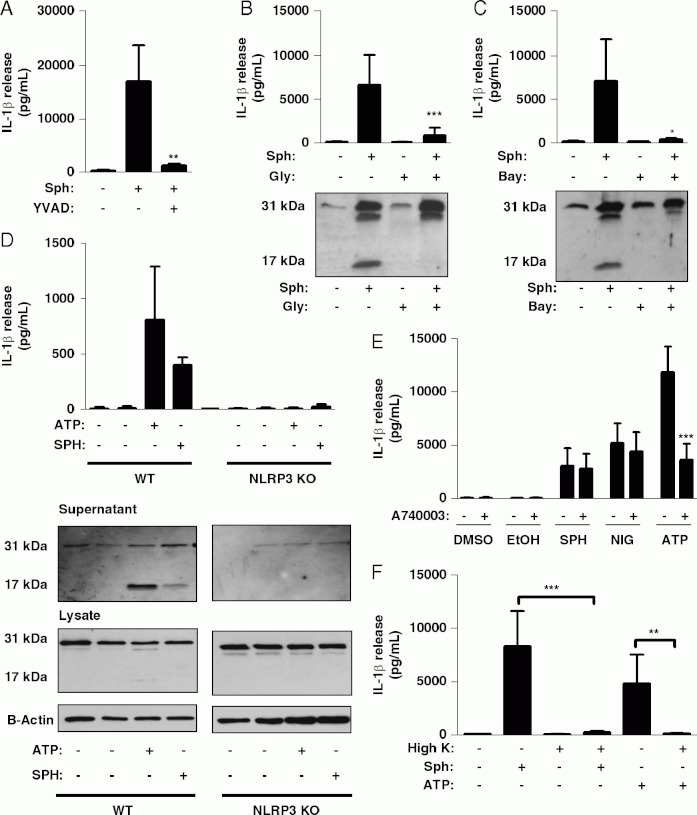
Sphingosine is a DAMP that activates the NLRP3-inflammasome. (A) LPS-treated (1mg/mL, 2h) murine peritoneal macrophages were incubated with Ac-YVAD-Cho (YVAD, 100 μM, 15 min) before Sph (20 μM, 1 h) or vehicle (0.5% DMSO) treatment. (B, C, E) LPS-treated macrophages were incubated with (B) glyburide (Gly, 100 μM, 15 min), (C) Bay 11-7082 (12 μM, 15 min), or (E) A740003 (10 μM, 15 min) before 0.5% DMSO, 1% ethanol (EtOH), Sph (20 μM, 1 h), ATP (5 μM, 15 min) or nigericin (NG, 20 μM, 15 min) treatment. (D) WTor NLRP3 KO macrophages were LPS treated (1 mg/mL, 2 h) and then incubated with vehicle (0.5% DMSO), Sph (20 μM, 1 h), or ATP (5 μM, 15 min). (F) LPS-treated macrophages were incubated in normal (5mM) or high K^1^ (150 μM) buffer and then incubated with vehicle (0.5% DMSO), Sph (20 μM, 1h) and ATP (5mM, 10min). IL-1β released into the supernatant was quantified by ELISA and IL-1β processing was determined by western blot. Data are shown as the mean1SD of three experiments, except (D) which is two experiments. ***p<0.001, **p<0.01, *p<0.05. Differences between groups were identified using one-way ANOVA with post-hoc Bonferroni multiple comparison test. Blots are representative of three experiments.

Incubation of LPS-primed macrophages with the selective caspase-1 inhibitor Ac-YVAD-CHO completely inhibited Sph-induced IL-1β secretion confirming the effect was caspase-1 dependent (Fig. 2A). The cellular sensor for DAMPs is suggested to be NLRP3 [7], which upon stimulation forms multimeric inflammasome complexes activating caspase-1 [6]. Sph-induced IL-1β secretion was blocked by glyburide and by Bay 11-7082 (Fig. 2B and C), both reported to inhibit NLRP3 inflammasome activation [25,26]. We also compared the effects of Sph on LPS-primed macrophages isolated from WT and NLRP3 KO mice. Sph-induced IL-1β secretion was abolished in NLRP3-deficient macrophages, confirming that, like other DAMPs, Sph activates NLRP3 inflammasomes to induce the secretion of IL-1 b (Fig. 2D). Extracellular ATP acting via the P2X7 receptor induces NLRP3-inflammasome activation [27], and several pro-inflammatory lipid mediators are reported to modulate P2X7 activity [28,29]. In contrast to ATP, Sph-induced IL-1β secretion was not inhibited by the specific P2X7 inhibitor A740003 (Fig. 2E). Potassium ion efflux is thought to be a key step in the induction of NLRP3-inflammasome activation [30] and nigericin, a potassium ionophore, induces P2X7-receptor-independent release of IL-1β (Fig. 2E). Consistent with this, incubation of LPS-primed macrophages in media containing high potassium ion concentration completely inhibited Sph and ATP-induced IL-1β secretion (Fig. 2F).

**Fig 3:**
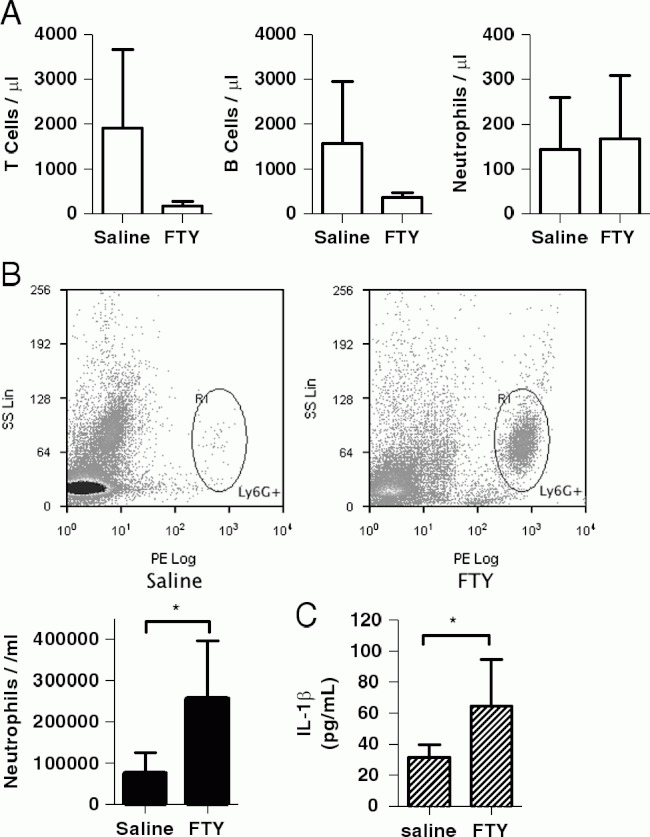
FTY720 acts like a DAMP in vivo. FTY720 (10mg/kg) or 0.9% saline was injected i.p. into C57BL6/J mice. After 6h, blood and a peritoneal lavage were collected. (A) Blood T cells (CD45^1^/CD3^1^), B cells (CD45^1^/B220^1^) and neutrophils (CD45^1^/Ly6G^1^) were quantified by flow cytometry. (B) CD45^1^/LY6G^1^ neutrophils in the peritoneal lavage were quantified by flow cytometry. (C) IL-1β in the lavage fluid was quantified by ELISA. Data are shown as the mean1SD of 3. *p<0.05. Differences between two groups were identified using the Student's t-test.

We next tested whether Sph could act as a DAMP in vivo using a model of peritonitis. Sph is very poorly soluble in aqueous solvents, and so to avoid injecting mice with high concentrations of DMSO, we injected mice i.p. with the water-soluble Sph analogue, FTY720 (FTY) at 10 mg/kg, a dose previously reported to induce tumour cell apoptosis and tumour regression [21,22]. Six hours after injection the peritoneum was lavaged and lavage fluid and blood were collected. Consistent with its known immunosuppressive effects [31], FTY720 reduced circulating T and B cells (Fig. 3A). This is due to phosphorylated FTY720, functionally antagonizing S1P receptors and causing lymphocyte sequestration in peripheral lymphoid organs as described above. However, FTY720 also induced significant IL-1β release (Fig. 3C) and neutrophil influx (Fig. 3B) into the peritoneal cavity, consistent with the pro-inflammatory effects observed for other DAMPs [32].

### Sph destabilizes lysosomes

**Fig 4:**
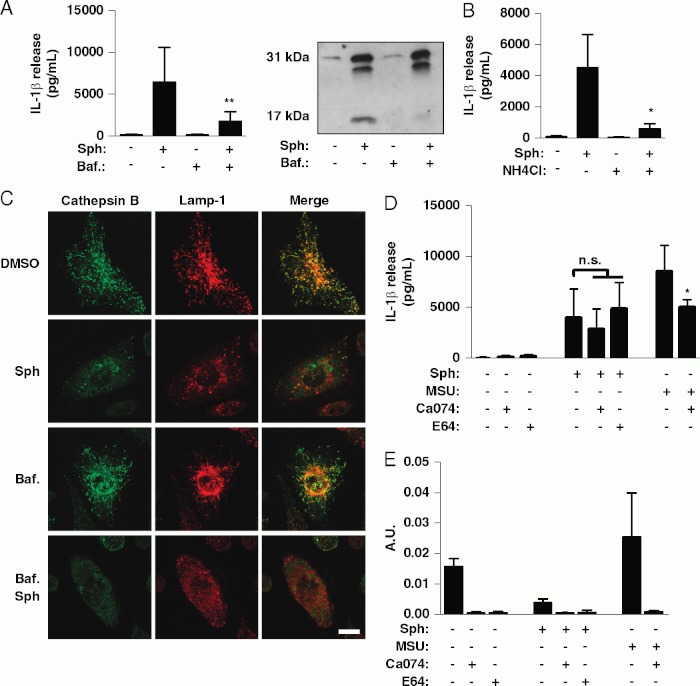
The role of lysosomal destabilization in Sph-induced IL-1β secretion. (A, B) LPS-treated (1 mg/mL, 2h) murine peritoneal macrophages were incubated with (A) bafilomycin (Baf., 100 nM, 15 min) or (B) ammonium chloride (NH4Cl, 20 μM, 15 min) before treatment with Sph (20 μM, 1 h) or vehicle (0.5% DMSO). IL-1β released into the supernatant was quantified by ELISA and IL-1β processing was determined by western blot. (C) LPS-treated macrophages were incubated with Baf. (100 nM, 15 min) before incubation with vehicle (0.5% DMSO) or Sph (20 μM, 30 min). The cells were then immunostained for the luminal lysosomal protease cathepsin B (green), and for the lysosomal membrane protein Lamp-1 (red). Scale bar 5 10 μM. Confocal images are representative of three cultures. (D, E) LPS-treated macrophages were incubated with Ca074-Me (50 μM, 15 min) or E64 (100 μM, 15 min) before treatment with Sph (20 μM, 1 h), MSU (250 mg/mL, 1 h) or vehicle (0.5% DMSO). (D) IL-1β released into the supernatant was quantified by ELISA and (E) cathepsin B/L activity in cell lysates was quantified using the fluorogenic cathepsin substrate Z-Phe-Arg-AMC. Data are shown as the mean1SD of three experiments. **p<0.01, *p<0.05. Differences between groups were identified using one-way ANOVAwith post-hoc Bonferroni multiple comparison test. Blots are representative of three experiments.

The destabilization of lysosomal membranes and lysosomal cathepsin activity is suggested to be a key point of integration for the activation of NLRP3 by structurally diverse DAMPs [10]. Increasing lysosomal pH by either blocking the vacuolar H^1^-ATPase with bafilomycin or by incubating cells with the lysosomotropic agent NH_4_Cl inhibited Sph-induced IL-1 b secretion (Fig. 4A and B). Incubation of LPS-primed macrophages with Sph (20 μM, 30 min) induced lysosomal membrane rupture, shown by the redistribution of cathepsin B from lysosomes to the cytosol (Fig. 4C). However, Sph-induced lysosomal membrane rupture did not correlate with IL-1 b secretion. Bafilomycin blocked IL-1β secretion (Fig. 4A) but did not block Sph-induced destabilization of lysosomal membranes (Fig. 4C). Furthermore, in contrast to MSU, Sph-induced IL-1β secretion was unaffected by either a cathepsin B (Ca074-Me) or a pan-cysteine protease inhibitor (E64, Fig. 4D). Both inhibitors blocked intracellular cathepsin activity as measured using a fluorogenic cathepsin B/L assay (Fig. 4E). These data suggest that lysosomal membrane destabilization per se is not sufficient to induce Sph-dependent-NLRP3-inflammasome activation, although there is a requirement for an acidic lysosome or compartment.

### Sph induced NLRP3-inflammasome activation requires a PP1/PP2A signal

**Fig 5:**
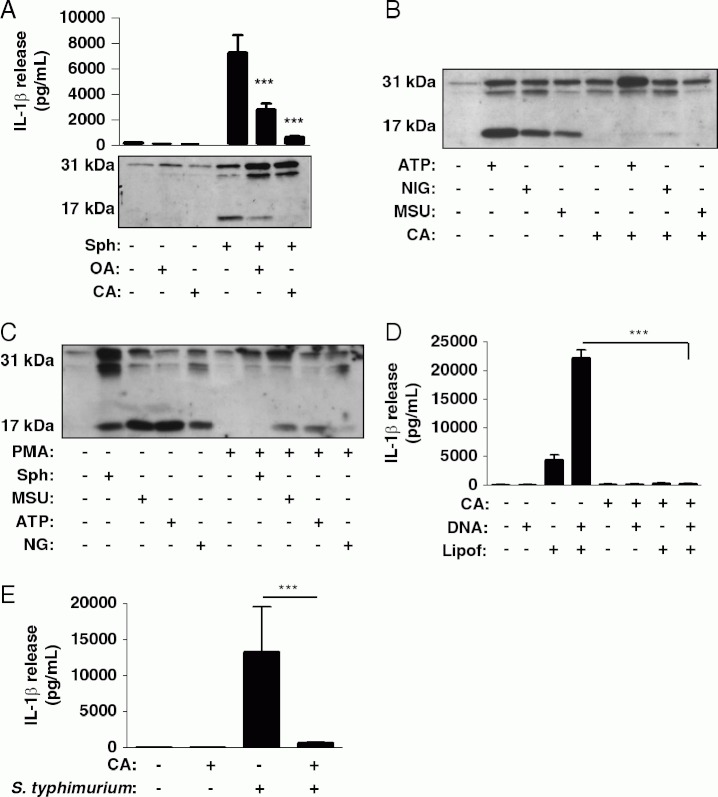
Sph-induced NLRP3-inflammasome activation requires a PP1/PP2A signal. (A) LPS-treated (1 mg/mL, 2h) murine peritoneal macrophages were incubated with okadaic acid (OA, 2 μM), calyculin A (CA, 50 nM) or 1% DMSO for 15 min before a 1-h incubation with Sph (20 μM) or 0.5% DMSO. Supernatants were collected and analyzed for IL-1β release and processing by ELISA and western blot. (B) LPS-treated peritoneal macrophages were incubated with calyculin A (CA, 50 nM) or 1% DMSO for 15 min before 15 min incubation with ATP (5 μM), nigericin (NIG, 20 μM), or 60 min with MSU (250 mg/mL). Supernatants were collected and analyzed for IL-1β processing and release by western blot. (C) LPS-treated peritoneal macrophages were incubated with PMA (500 nM) or 0.5% ethanol for 15 min before 15 min incubation with ATP (5mM), nigericin (NIG, 20 *mM),* or 60 min with MSU (250mg/mL). Supernatants were collected and analyzed for IL-1β processing and release by western blot. (D) LPS-treated macrophages were incubated with lipofectamine, DNA or lipofectamine-DNA complexes for 5h in the absence and presence of calyculin A (CA, 50 nM). IL-1β released into the supernatant was quantified by ELISA. (E) LPS-treated macrophages were infected with typhimurium (SL1344, MOI 30) for 3 h in the absence and presence of calyculin A (CA, 50 nM, 15 min pre-treatment). IL-1β released into the supernatant was quantified by ELISA. Data are shown as the mean1SD of three experiments ***p<0.001. Differences between groups were identified using one-way ANOVA with post-hoc Bonferroni multiple comparison test. Blots are representative of three experiments.

HS Sph and FTY720 are known to induce an increase in the activity of the phosphatase PP2A [33,34]. To test whether Sph-induced NLRP3- and caspase-1-activation occurred via a similar mechanism, we incubated LPS-treated macrophages with the PP1 and PP2A serine/threonine phosphatase inhibitors calyculin A (CA) and okadaic acid (OA) just prior to incubation with Sph. Both inhibitors blocked the release of IL-1β (Fig. 5A). CA also completely inhibited IL-1β release induced by the other NLRP3-inflammasome activators ATP, nigericin and MSU (Fig. 5B). Phorbol 12-myristate 13-acetate (PMA) is an activator of members of the PKC family, ubiquitous serine/threonine kinases [35]. Treatment of LPS-primed peritoneal macrophages with PMA inhibited the release of IL-1β in response to Sph, ATP, MSU and nigericin (Fig. 5C). These data suggest that structurally diverse activators of a NLRP3 inflammasome require a PP1/PP2A signal. We then sought to determine whether a PP1/PP2A signal was specific for the NLRP3 inflammasome or whether it represented a mechanism regulating the control of inflammasomes in general. An NLRP3-independent activation of caspase-1 occurs following transfection of macrophages with DNA [36], and which is dependent upon the AIM2 inflammasome [37,38]. We therefore tested the effects of CA on inflammasome activation caused by DNA transfection and found that it completely inhibited IL-1β release (Fig. 5D). Infection of macrophages with the pathogen *typhimurium* is known to activate the NLRC4 inflammasome [39]. CA also inhibited *S. thyphimurium* -induced IL-1β release (Fig. 5E). These data suggest that a PP1/PP2A signal is essential for the activation of multiple inflammasomes.

## Discussion

IL-1 contributes to the pathogenesis of diverse diseases, and thus understanding the mechanisms of its production may identify new therapeutic targets [3]. We have discovered that the bioactive lipid metabolite Sph can act as a DAMP. In vitro it induced NLRP3-dependent activation of caspase-1 and secretion of IL-1β. We have shown that an Sph analogue, FTY720, induced IL-1β secretion from LPS-primed peritoneal macrophages in vitro, and also that it induced IL-1β release and neutrophil influx in an in vivo model of peritonitis, hallmarks of DAMPs such as MSU and DAMPs released from dead cells [32,40]. Sph levels are elevated during disease [15–17], and thus Sph could act as a DAMP when released from dying cells. Alternatively, intracellular Sph production and signalling by macrophages at sites of inflammation and tissue injury may regulate NLRP3-inflammasome activation, since we know that inhibitors of acid sphingomyelinase inhibit ATP-induced release of IL-1β from glial cells [41].

Sph induces apoptotic and necrotic cell death, an effect that is attributed to its ability to accumulate in lysosomes and destabilize their membranes, leading to a translocation of destructive lysosomal proteases in to the cytosol [18]. Destabilization of lysosomal membranes and cathepsin activity is also important for NLRP3 inflammasome activation in response to a number of stimuli [12,13]. It was based on these observations that we hypothesized that Sph-dependent lysosomal membrane disruption would explain the effect on IL-1β release. However, despite bafilomycin A and NH4Cl inhibiting Sph-induced IL-1β release (Fig. 4), this effect was independent of cathepsin activity, and of lysosomal membrane destabilization (Fig. 4). Thus although the effects of Sph on IL-1β release seem dependent upon an acidified lysosome, they are independent of the lysosomal mechanisms that regulate IL-1β release that have been described in the literature to-date. This alternative mechanism could depend upon the activity of another protease or hydrolase, a trafficking process or even the dissociation of a ligand from a receptor following its endocytosis, although this is something that requires further investigation.

Both Sph and FTY720 are known to activate PP2A [33,34] and using the inhibitors CA and OA, we identified this as an important step in Sph-induced NLRP3-inflammasome activation and IL-1β release (Fig. 5). We then extended this finding to show that a PP1/PP2A-dependent signal was important for NLRP3 activation in general, since CA inhibited the release of IL-1β induced by ATP, nigericin and MSU. CA also blocked IL-1β release in response to activators of the AIM2 and NLRC4 inflammasomes (Fig. 5), suggesting that a PP1/PP2A-dependent signal is important for the activation of inflammasomes in general. There are 428 serine/threonine kinases in the human genome yet only approximately 30 serine/threonine phospha-tases that control the phosphorylation of thousands of protein substrates [42]. PP2A is a trimeric holoenzyme, and specificity for PP2A to specific processes or cellular locations is conferred by the interaction of the catalytic subunit (PPP2CA and PPP2CB), one of two scaffold subunits (PPP2R1A or PPP2R1B), and a regulatory subunit, for which there are at least 26 alternatively spliced forms from 15 genes [42–44]. Thus there is huge complexity in this system. However, given that here we have reported a diverse and wide range of stimuli that activate multiple inflammasomes all requiring a PP1/PP2A-dependent signal, it would suggest that the phosphatase-dependent signal is at a late stage of inflammasome activation, where the common elements are ASC, caspase-1 and pro-IL-1β.

In summary, we have shown that the endogenous bioactive lipid metabolite Sph can activate the NLRP3 inflammasome in vitro, and exhibits typical behaviour of a DAMP when injected in vivo. These data provide fresh insights into the potential role of lipid metabolites in inflammation and highlights an additional effect of Sph that has, up to now, been unreported. We also report that the Sph effect is dependent upon dephosphorylation of a serine/threonine residue since the PP1/PP2A inhibitors CA and OA inhibited Sph-induced IL-1β release. A role for a PP1/PP2A-dependent signal was found for other NLRP3-inflammasome activators, and also for the activation of the AIM2 and NLRC4 inflammasomes. These data suggest that this common phosphatase-dependent signal is occurring at the latter stages of inflammasome assembly where some of the components (e.g. ASC, caspase-1 and IL-1) are common.

## Materials and methods

### Mice

NLRP3∼^7^∼ mice were generously provided by Dr. Vishva Dixit, Genentech [27,39]. C57BL/6J mice were purchased from Harlan. In vivo experiments were performed under appropriate UK Home Office personal and project licences using protocols which adhered to the UK Animals (Scientific Procedures) Act 1986.

### Reagents

Bacterial lipopolysacharide (LPS, *Escherichia coli* 026:B6), synthetic D-sphingosine and all other chemicals were from Sigma unless stated otherwise. Foetal bovine serum (FBS) was from PAA Laboratories. Bay 11-7082 was from Enzo Life Sciences. Ac-YVAD-CHO, E-64, DMS, Ca074-Me and Z-Phe-Arg-AMC were from Merck Chemicals (2R,4R)-4-aminopyrrolidine (APDC), ceramide and A740003 were from Tocris Bioscience. FTY720 and FTY720-P were from Santa Cruz. MSU crystals were from Invivogen. Donkey serum was from Stratech Scientific. The anti-mouse IL-1β antibody used for Western blot (S329) was a kind gift from the National Institute of Biological Standards and Controls. The cathepsin B antibody and the Lamp-1 antibody were from R&D Systems and Abcam, respectively. Alexa Fluor secondary antibodies and ProLong Gold with DAPI were from Invitrogen. The allophycocya-nin-conjugated anti-CD45 antibody was from eBiosciences, PE-conjugated anti-Ly6G and PE-conjugated anti-B220 were from BD Pharmingen and FITC-conjugated anti-CD3 was from AbD Serotec. FACS lysis solution was from BD Biosciences.

### Cell culture

Macrophages were prepared from adult, male C57BL/6 mice (supplied by Harlan, UK), as described previously [45]. Cultured peritoneal macrophages were LPS-primed (1 mg/mL, 2 h) to induce pro-IL-1β expression. The cells were then treated with inhibitors as indicated for 15min, prior to addition of the sphingolipid metabolites at the indicated concentrations for 1 h and collection of supernatants for analysis of IL-1β processing and release. ATP (5mM, 10min), nigericin (20mM, 15min) and MSU (250 mg/mL, 1 h) treatments were also used. *S. typhimurium* (strain SL1344, MOI 30) was used to infect LPS-treated macrophages in serum- and antibiotic-free media for 3 h prior to the collection of supernatants. For the AIM2 activating experiments, endotoxin-free plasmid DNA (empty mammalian expression vector pCMV-Tag4B) was prepared using a Qiagen EndoFree plasmid MAXI kit. After LPS-treatment, macrophages were transferred to serum- and antibiotic-free media, and treated with inhibitors immediately before transfection with pCMV-Tag4B using lipofectamine according to the manufacturers' instructions. Control wells were treated with DNA or lipofectamine alone. Cells were incubated for 5h before the collection of supernatants for analysis of IL-1β release.

### ELISA

Macrophage supernatants, plasma, lavage fluid and lavage cell lysates were analyzed for IL-1a and b using specific ELISA kits from R&D Systems, used according to manufacturer's instructions.

### Western blot

Macrophage supernatants were harvested and prepared in sample buffer containing 1% b-mercaptoethanol. Samples were boiled and then electrophoresed on 12% SDS-acrylamide gels. Proteins were transferred to a nitrocellulose membrane and blotted with a sheep anti-mouse IL-1β polyclonal serum followed by an HRP-conjugated goat anti-sheep antibody, and subsequently detected using enhanced chemi-luminescence reagents (ECL, GE Healthcare, UK).

### Immunocytochemistry and microscopy

Following bafilomycin/Sph treatment, macrophages on glass coverslips were fixed with 4% paraformaldehyde (PFA) in PBS. The cells were permeabilized with 0.1% Triton-X100 and then quenched with 0.25% ammonium chloride. A blocking step for 1 h using 5% BSA/5% donkey serum (block solution) was used prior to incubation with the goat anti-cathepsin B or rat anti-lamp-1 antibodies in block solution for 1 h. Coverslips were then washed in PBS. Primary antibodies were detected by incubation with Alexa Fluor 488-conjugated donkey anti-goat and Alexa Fluor 594-conjugated donkey anti-rat antibodies in blocking solution for 1 h. The coverslips were washed again with PBS and mounted onto a glass slide using ProLong Gold mounting medium containing DAPI. Confocal microscopy on fixed cells was performed using a Leica TCS SP5 AOBS confocal microscope (63 x/1.40 HCX PL Apo objective) with the Leica LAS AF software.

### Cathepsin B/L assay

Cells were lysed in hypotonic lysis buffer (25 μM HEPES, 5 μM EGTA, 5mM DTT, pH 7.5) on ice, and lysates were mixed with 2 x reaction buffer (0.2 M sodium acetate buffer, 4mM EDTA, 4mM DTT, pH 5.5). Cathepsin B/L-dependent cleavage of the fluorogenic substrate Z-Phe-Arg-AMC (40 μM) was measured by an increase in fluorescence (excitation 335 nm, emission 460 nm).

### Peritonitis model

C57BL/6J mice were injected intraperitoneally (i.p.) with FTY720 (10mg/kg) or 0.9% saline (mice were randomized to treatment groups and investigators were blinded to groups). Mice were killed 6h post-injection, blood samples were taken via cardiac puncture and the peritoneal cavity was lavaged with PBS with 0.1% BSA and 1 μM EDTA. Lavage cells were labelled with Allophycocyanin-anti-CD45 and PE-anti-Ly6G. The proportion of CD45^1^/Ly6G^1^ neutrophils in the lavage was determined by flow cytometry (CyAn ADP Flow Cytometer, Dako, with Summit 4.0 analysis software). Absolute neutrophil numbers were calculated using a total leucocyte count (Coulter Counter, Beckman Coulter). Blood cells were labelled with APC-anti-CD45 with either PE-anti-Ly6G or PE-anti-B220 and FITC-anti-CD3. Red blood cells were lysed with FACS lysing solution, and AccuCheck counting beads were added to enable cell number determination. Absolute numbers of neutrophils, CD45^1^/CD3^1^ T cells and CD45^1^/B220^1^ B cells were determined by flow cytometry (see Supporting Information Fig. 1 for the gating strategy used). IL-1β in plasma, peritoneal lavage cell lysates and lavage fluid was quantified by ELISA.

### Statistical analysis

Statistical analyses were performed using the GraphPad Prism version 5.00 for Windows (GraphPad Software, www.graphpad.com). Differences between two groups were identified using the Student's t-test, and differences between three or more groups were identified using one-way ANOVA with post-hoc Bonferroni multiple comparison test. All data are expressed as mean 7 standard deviation (SD). ***p<0.001, **p<0.01, *p<0.05.
