# Supplementary material for: Sphingosine regulates the NLRP3-inflammasome and IL-1β release from macrophages
Source: Eur J Immunol. 2011 Dec 27;42(3):716–25. doi: 10.1002/eji.201142079 (PMC3491674; doi:10.1002/eji.201142079)
Supplement: Supplementary file 1 [file eji0042-0716-SD1.pdf]

European Journal of Immunology

Supporting Information

for

DOI 10.1002/eji.201142079

Sphingosine regulates the NLRP3-inflammasome and IL-1β release from macrophages

Nadia M. Luheshi, James A. Giles, Gloria Lopez-Castejon and David Brough

Supporting information Figure 1

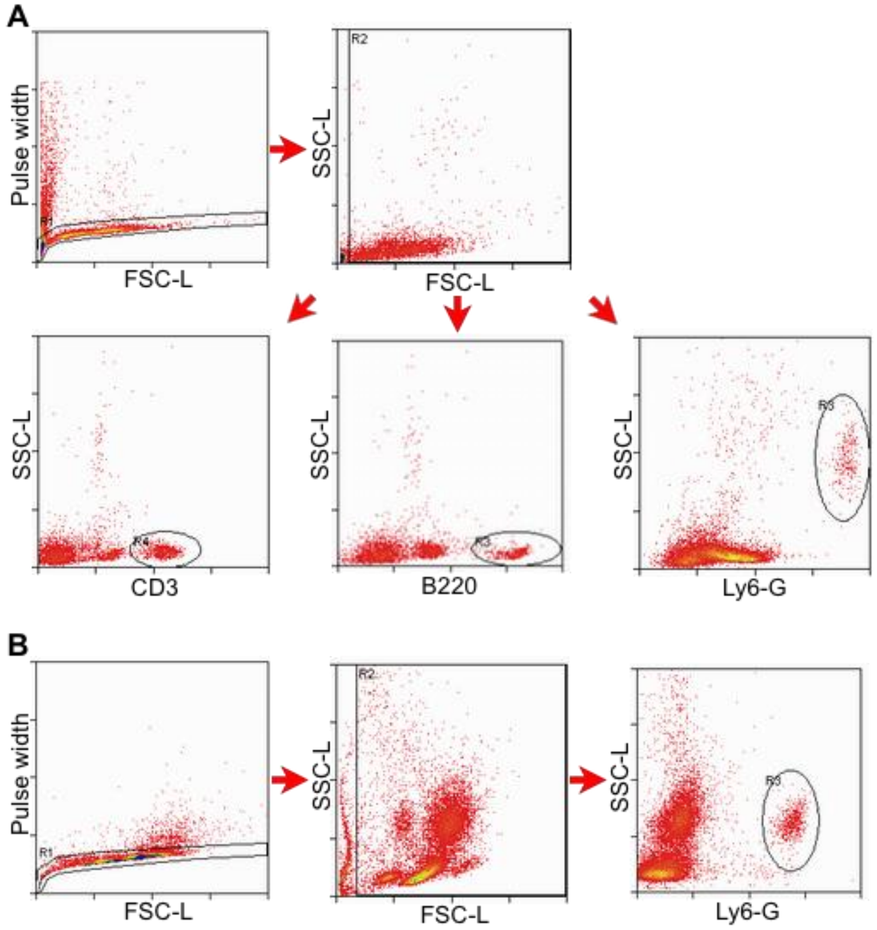

Representative gating strategies for: **A.** *In vivo* experiments analysing the effect of FTY720 injection on T cell (CD3+), B cell (B220) and neutrophil (Ly6G+) numbers in blood. **B.** *In vivo* experiments analysing the effect of FTY720 injection on neutrophil (Ly6-G+) numbers in peritoneal lavage fluid.
